# Siltuximab (CNTO 328) with lenalidomide, bortezomib and dexamethasone in newly-diagnosed, previously untreated multiple myeloma: an open-label phase I trial

**DOI:** 10.1038/bcj.2016.4

**Published:** 2016-02-12

**Authors:** J J Shah, L Feng, S K Thomas, Z Berkova, D M Weber, M Wang, M H Qazilbash, R E Champlin, T R Mendoza, C Cleeland, R Z Orlowski

**Affiliations:** 1 Department of Lymphoma and Myeloma, The University of Texas MD Anderson Cancer Center, Houston, TX, USA; 2 Department of Biostatistics, The University of Texas MD Anderson Cancer Center, Houston, TX, USA; 3 Department of Stem Cell Transplantation, The University of Texas MD Anderson Cancer Center, Houston, TX, USA; 4 Department of Symptom Research, The University of Texas MD Anderson Cancer Center, Houston, TX, USA; 5 Department of Experimental Therapeutics, The University of Texas MD Anderson Cancer Center, Houston, TX, USA

## Abstract

The safety and efficacy of siltuximab (CNTO 328) was tested in combination with lenalidomide, bortezomib and dexamethasone (RVD) in patients with newly-diagnosed, previously untreated symptomatic multiple myeloma. Fourteen patients were enrolled in the study, eleven of whom qualified to receive therapy. A majority of patients (81.8%) completed the minimal number or more of the four required cycles, while two patients completed only three cycles. The maximum tolerated dose (MTD) of siltuximab with RVD was dose level −1 (siltuximab: 8.3 mg/kg; bortezomib: 1.3 mg/m^2^; lenalidomide: 25 mg; dexamethasone: 20 mg). Serious adverse events were grade 3 pneumonia and grade 4 thrombocytopenia, and no deaths occurred during the study or with follow-up (median follow-up 28.1 months). An overall response rate, after 3–4 cycles of therapy, of 90.9% (95% confidence interval (CI): 58.7%, 99.8%) (9.1% complete response (95% CI: 0.2%, 41.3%), 45.5% very good partial response (95% CI: 16.7%, 76.6%) and 36.4% partial response (95% CI: 10.9%, 69.2%)) was seen. Two patients withdrew consent, and nine patients (81.8%) opted for autologous stem cell transplantation.

## Introduction

Multiple myeloma (MM) is defined by the clonal expansion of malignant plasma cells, and is the second most commonly diagnosed hematological malignancy.^1^ More than 24 000 new myeloma cases are diagnosed in the United States each year, and 114 000 worldwide (http://globocan.iarc.fr/Default.aspx). It is estimated that, due in part to an aging population, the incidence of cancer including myeloma will increase by almost 45% in the next 20 years.^2^

Development and regulatory approval of new classes of drugs such as immunomodulatory agents (thalidomide, lenalidomide and pomalidomide), histone deacetylase inhibitors (panobinostat) and proteasome inhibitors (bortezomib, carfilzomib) have led to paradigm shifts in the approach and treatment options for patients with MM. These novel therapies have contributed to increases in the overall survival from 2–3 years to 5–7 years currently.^3, 4^ Bortezomib and lenalidomide were shown to induce apoptosis in myeloma cells by different but partially overlapping mechanisms, and their combination produced a synergistic killing effect.^5, 6, 7^ The combination of proteasome inhibitors and immunomodulatory agents induced high response rates and complete remissions (CRs) in several clinical trials.^8, 9^ In particular, the combination of lenalidomide, bortezomib and dexamethasone (RVD) demonstrated a significant efficacy in both newly-diagnosed and relapsed or refractory MM.^8, 10^ However, the CR rates with limited courses of induction therapy before consolidation with an autologous stem cell transplant remain <25%. There are also increasing data which demonstrate that improved depths of response, including the achievement of CR, stringent CR and minimal residual disease-negative status, translate to improvements in survival and outcomes.

Interleukin 6 (IL-6) is known to enhance proliferation and survival of cancer cells, including malignant plasma cells, and elevated IL-6 levels in the serum are associated with poor prognosis in myeloma.^11, 12, 13^ IL-6 was shown to protect MM cells from apoptosis induced by steroids and chemotherapeutic drugs, including bortezomib. Klein *et al.* showed biological activity of blocking IL-6 with an anti-IL-6 monoclonal antibody in 10 patients with relapsed/refractory MM and plasma cell leukemia, with reduction in proliferative indices, though no responses were seen.^14^ Pre-clinical studies demonstrated that the combination of siltuximab and bortezomib had a potentially synergistic effect in inducing apoptosis in both IL-6-dependent and IL-6-independent MM cell lines. This effect was preserved in the presence of bone marrow stromal cells, and in CD138^+^ myeloma samples derived from patients with relative clinical resistance to bortezomib. Additionally, blocking IL-6 can also increase the effectiveness of both steroids and lenalidomide in treating myeloma.^15, 16, 17^ Blocking IL-6 by siltuximab, a chimeric anti-IL-6 monoclonal antibody formerly known as CNTO 328, showed therapeutic efficacy in inflammatory diseases and multicentric Castleman's disease with a favorable safety profile.^18^ Attempts to improve depths of response and CR rates with induction therapy have included strategies to incorporate a fourth agent to the combination of lenalidomide/bortezomib/dexamethasone (RVD), such as anthracyclines and alkylators, with limited success and increased toxicity. We hypothesized that the addition of siltuximab to the RVD regimen could improve response rates with a limited increase in toxicity. We report here the results of an open-label, phase I/II study to assess the safety and efficacy of siltuximab combined with RVD in newly-diagnosed, previously untreated symptomatic myeloma patients.

## Subjects and methods

### Patients

Patients with a confirmed diagnosis of MM by the IMWG (International Myeloma Working Group) Criteria^19^ were eligible for the study if they had received no prior systemic myeloma therapy. Prior local radiotherapy, with or without concomitant exposure to steroids for pain control or management of cord/nerve root compression, was allowed, and patients were required to register into, and comply with the RevAssist program. Additional key inclusion criteria included: (1) Eastern Cooperative Oncology Group performance status of 0–2 and (2) age>18. Key exclusion criteria included: (1) patients with greater than grade 2 peripheral neuropathy; (2) renal insufficiency (creatinine clearance <30 ml/min by the Cockroft-Gault formula); (3) mucosal or internal bleeding and/or inability to maintain a platelet count of ⩾50 000 cells/mm^3^; (4) absolute neutrophil count <1000 cells/mm^3^ without growth factors; (5) bilirubin >1.5 mg/dl; (6) hemoglobin <8.0 g/dl, with transfusions permitted; (7) aspartate and alanine aminotransferase ⩾2 × the upper limit of normal; (8) myocardial infarction within 6 months before enrollment, New York Heart Association Class III or IV heart failure, uncontrolled angina, severe uncontrolled ventricular arrhythmias, electrocardiographic evidence of acute ischemia or active conduction system abnormalities (any electrocardiographic abnormality at screening or Cycle 1 Day 1 had to be documented by the investigator as not medically relevant); (9) clinically relevant active infection requiring intravenous antibiotics; (10) serious co-morbid medical conditions such as uncontrolled chronic obstructive or chronic restrictive pulmonary disease, cirrhosis, uncontrolled diabetes mellitus (fasting blood sugar >400 mg/dl despite medical treatment); (11) known history of polyneuropathy, organomegaly, endocrinopathy, monoclonal gammopathy and skin changes syndrome; and (12) active human immunodeficiency virus, hepatitis B or hepatitis C infection. Those vaccinated with any live, attenuated vaccine within 4 weeks of the first dose of study treatment, and pregnant or breast-feeding female subjects were also excluded.

The trial was approved by the MD Anderson Institutional Review Board, and all patients provided written informed consent before study enrollment. This study was conducted in accordance with the Declaration of Helsinki and the guidelines for Good Clinical Practice, and was registered with Clinicaltrials.gov: identifier NCT01531998.

### Study design and treatment

This was an open-label, phase I/II, multicenter study in patients with newly-diagnosed MM evaluating the combination of RVD and siltuximab. The primary objective of the phase I was to determine the recommended dose of siltuximab in combination with RVD.^20^ The primary objective of the phase II was to evaluate the CR/near CR (nCR) rate of the combination of siltuximab with RVD after eight cycles of therapy. Additional key secondary objectives included to: (1) define the overall response rate (stringent CR/nCR/very good partial response (VGPR)/partial response (PR)) after eight cycles; (2) evaluate tolerability and toxicity; (3) examine the effect on the number of CD34^+^ cells (per kg) collected, and the days of harvest required, as well as engraftment parameters as exploratory end points for patients who proceeded on to stem cell transplantation.

Patients treated at dose level 1 received lenalidomide 25 mg administered orally daily on days 1–14, followed by 7 days of rest every 21 days; bortezomib 1.3 mg/m^2^ subcutaneously on days 1, 4, 8 and 11; dexamethasone 20 mg on days 1, 2, 4, 5, 8, 9, 11 and 12; and siltuximab 11 mg/kg iv on day 1. Siltuximab dosing was decreased to 8.3 mg/kg at dose level −1. Patients were initially planned to receive treatment for four cycles of induction therapy, but were allowed to proceed to stem cell harvest after two cycles with the mobilization regimen based on physician's discretion. After four cycles of induction therapy patients were eligible for autologous stem cell transplant (ASCT). If a delayed transplant option was chosen, then patients continued with induction therapy at least two cycles beyond achieving their best response for a minimum of four cycles of therapy, and a maximum of eight cycles of therapy, and then transitioned to a maintenance regimen. Maintenance consisted of lenalidomide at the last tolerated dose on days 1–21 every 28 days; the siltuximab dose recommended from phase 1 administered q28 days; and dexamethasone 20 mg once weekly. Patients remained on study until disease relapse, unacceptable toxicity, withdrawal of consent, or no further clinical benefit was experienced at the discretion of treating physician.

### Response and toxicity assessment

Efficacy and toxicity were evaluated after each cycle of therapy, and patients with progressive disease or intolerable toxicities were removed from the trial. Responses were assessed after completion of each cycle using the EBMT (European Group for Blood and Marrow Transplantation) Response Criteria^21^ and the IMWG criteria.^22^ Full response assessments were to be performed after eight cycles of therapy, or at the time when the patient proceeded to ASCT. Toxicity was assessed after each cycle of therapy and graded according to the National Cancer Institute‘s Common Terminology for Adverse Events, version 4.0. Dose limiting toxicity (DLT) was defined as any non-hematological toxicity grade 3 or higher occurring in the first cycle except: (1) nausea/vomiting that responded to systematic therapy or (2) alopecia. Hematological DLTs were determined based on the first cycle, and included: (1) grade 4 neutropenia lasting more than 5 days; (2) febrile neutropenia (absolute neutrophil count <1.0 × 10^9^/l, fever ⩾38.5 °C) of any duration (filgrastim was allowed after a DLT was recorded); (3) grade 4 thrombocytopenia; or (4) grade 3 thrombocytopenia with bleeding, or any requirement for platelet transfusion.

The MD Anderson Symptom Inventory (MDASI), a brief, validated patient-reported outcome tool to measure 13 myeloma and cancer-related symptoms,^23^ was used to evaluate the effect of the combination therapy on symptom burden, and the impact of these symptoms on daily functioning. The symptoms to be assessed by the core MDASI-MM module included: pain, fatigue, nausea, disturbed sleep, distress, shortness of breath, difficulty remembering, lack of appetite, drowsiness, dry mouth, sadness, vomiting, numbness, constipation, muscle weakness, diarrhea, mouth or throat sores, rash, and trouble concentrating. Six additional items assessing symptom-related interference in general activity, mood, work, relation with others, enjoyment of life, and walking were included as well. Data were collected at baseline and day 1 of every treatment cycle.

### Statistical methods

The standard '3+3' design was used with two dose levels of siltuximab, as shown in Table 1. Applying the 3+3 design, the first cohort of three patients was treated at dose level 1 and evaluated for DLTs at the end of the first cycle. At any given dose, if greater than one out of three patients or one out of six patients experienced DLTs, then that dose level was defined as exceeding the maximum tolerated dose (MTD). Summary statistics, including median and range for continuous variables, frequency counts and percentages for categorical variables, were provided. Response rate and its 95% confidence interval were calculated.

## Results

### Patient characteristics

Fourteen newly-diagnosed MM patients were screened and enrolled at The University of Texas MD Anderson Cancer Center. Three patients failed screening studies and did not receive protocol directed therapy, while eleven patients received at least one cycle of therapy. The median age of the 11 treated patients was 62 years (range, 47–73). Most patients were female, Caucasian, had stage I disease and had a normal diploid karyotype (Table 2).

### Treatment toxicity and MTD

Toxicity was evaluated in all 11 treated patients after each cycle of therapy, and 2 out of 6 who received treatment at dose level 1 (siltuximab 11 mg/kg) experienced DLTs. The first patient was hospitalized for a non-neutropenic fever, received 1 day of intravenous antibiotics, which were subsequently transitioned to oral antibiotics, and therefore was defined as having grade 3 pneumonia. The second DLT was a transient grade 4 thrombocytopenia with a platelet count of 24 K/μl on day 15 that quickly recovered to 54 K/μl on day 19, and 137 K/μl on day 22 of therapy. Among the five patients who received siltuximab at dose level −1 (8.3 mg/kg), there were no DLTs encountered. The MTD allowed one DLT, and therefore as no DLTs were encountered in the first five patients, even if a sixth patient was treated and encountered a DLT the MTD of siltuximab in combination with RVD would still have been defined as 8.3 mg/kg.

### Adverse events

There was no unexpected toxicity and no deaths were encountered during this trial. The observed grade 4 hematological adverse events (AEs) were neutropenia and thrombocytopenia, while the most frequent grade 3–4 hematological AEs were grade 3 lymphopenia, leukopenia, and neutropenia (Table 3). There were no grade 4 non-hematological AEs, while grade 3 non-hematological AEs were peripheral sensory neuropathy, pneumonia, maculo-papular rash, edema of the limbs, nausea, and diarrhea (Table 3). One patient withdrew from the study due to grade 2 peripheral neuropathy with pain and grade 3 lower extremity edema after completion of three cycles of therapy. In addition, one patient had treatment cycles 2 and 4 delayed due to grade 3/4 neutropenia.

Treatment had no adverse impact on stem cell mobilization, and adequate stem cells were collected from all patients, as shown in Table 4. The median number of stem cells collected was 4.16 × 10^6^ CD34^+^ cells (range: 2.88–19.22). After transplant, the median day of engraftment was 11 (range: 11–12).

### Treatment efficacy

Treatment outcomes are summarized in Table 5. A majority of patients (9/11) completed the required minimum of four cycles of induction therapy. One patient entered a CR after cycle 3 and proceeded to ASCT; one patient withdrew consent after cycle 3 as described above due to G3 PN. In all, 2 of 11 treated patients withdrew from the study and the remaining 9 patients chose to proceed to ASCT after completion of 3–5 cycles of therapy (Table 5).

After four cycles of therapy, the overall response rate (⩾PR) was 90.9% (10/11), including four patients with PR, five with VGPR and one patient with CR. One patient had a minor response. The response rates at 3 and 6 months after ASCT are listed in Table 5. There was a further improvement in response in 5/8 patients, while 3 patients who achieved a PR before transplant remained in a PR post-transplant.

Among nine patients in active follow-up (median follow-up 23.8 months (725 days); range: 6.7–28.3 (203–862)), all remain in remission post-transplant; one patient was lost to follow-up; and one withdrew consent.

### Quality of life: MDASI

Among the 11 patients, there were a total of 43 MDASI measurements at cycle 1, day 1 (C1D1), C2D1, C3D1, and C4D1. The overall top five most severe symptoms were fatigue, numbness, pain, disturbed sleep, and constipation. Average symptom scores of the 11 patients did not show significant changes over time. Over the 4 time points, 8 of the 11 patients were asymptomatic (scoring 0–3 on a 0–10 scale), while 3 reported moderate/severe (scoring 4 or greater) symptom burden. One patient reported severe fatigue (score=8), pain (score=9), and disturbed sleep (score=8) at C1D1, and kept reporting high levels of fatigue and pain at the following time points, however, disturbed sleep resolved over time. Two patients started without symptoms and reported increased fatigue, pain, numbness, disturbed sleep and/or constipation over time.

## Discussion

We report here the safety and efficacy of the combination of siltuximab and RVD in patients with newly-diagnosed symptomatic myeloma, and conclude that the MTD of siltuximab was 8.3 mg/kg in this regimen. A previous phase 1 dose escalation of siltuximab as a single agent administered every 2–3 weeks established 12 mg/kg as a dose without DLTs and with clinical activity, including CR in 2/13 patients.^24^ A phase 2 study of single-agent siltuximab in relapsed/refractory myeloma administered siltuximab at 6 mg/kg on days 1 and 15 of a 28- day cycle.^25^ Moreover, a phase II study of bortezomib-melphalan-prednisone in combination with siltuximab administered this antibody at 11 mg/kg every 3 weeks in patients with newly-diagnosed myeloma.^26^ Finally, siltuximab dosed at 6 mg/kg every 2 weeks was also combined with bortezomib in relapsed/refractory disease.^27^ Thus, the dose identified here of 8.3 mg/kg every 3 weeks is within the range that has been previously studied for siltuximab as a single agent or in combination. These data, in combination with previously published experience, suggest that siltuximab can be combined safely with various anti-myeloma regimens.

The toxicities encountered at 11 mg/kg, including grade 3 pneumonia and grade 4 thrombocytopenia, can be expected toxicities associated with RVD. The addition of siltuximab was therefore not associated with a more prolonged or severe toxicity, and no DLTs were encountered at a siltuximab dose of 8.3 mg/kg. There is an increased incidence of infections associated with siltuximab, as 29% grade ⩾3 infections were observed when combined with bortezomib-melphalan-prednisone (VMP) compared with only 17% with VMP alone, and 62% when siltuximab was combined with bortezomib versus 49% for bortezomib alone. However, in our trial, only one patient had grade 3 pneumonia, and increased infectious complications were not seen compared with previous experiences with siltuximab. There were no on-study deaths and no unexpected toxicities. Importantly, this was the first experience with siltuximab in newly-diagnosed, transplant eligible patients as induction therapy before ASCT, and there was no effect on stem cell mobilization or subsequent autologous stem cell transplant/engraftment.

The combination was highly active, with a 91% response rate at 4 cycles, including 1 patient in CR, and 6/11 patients (54.5%) with response ⩾VGPR after a brief course of therapy. Notably, this VGPR rate was higher than what would have been expected for RVD alone,^28^ suggesting the possibility that targeting IL-6 could remain an interesting approach to myeloma therapy. However, this could also be due to the high proportion of patients with ISS stage I disease. There was continued improvement in the depth of response in 5/8 patients who proceeded to ASCT, and all patients in follow-up continue to remain in remission. However, the efficacy data are limited by the small number of patients since the trial was halted and did not proceed to phase II. This was due to the negative outcomes from the phase II study of VMP vs VMP+siltuximab, which demonstrated no significant improvement in progression-free survival, at which point further development of siltuximab in symptomatic myeloma was halted by the sponsor.

Finally, incorporation of a patient-reported outcome tool such as the MDASI is an important aspect of clinical trial design. We demonstrated the feasibility of obtaining patient-reported outcome on a relatively intense schedule (on day 1 of each cycle), and identified the most common symptoms to be fatigue, numbness, pain, disturbed sleep, and constipation. Importantly, there was no significant change in symptom burden over the first four cycles, and only two patients described increased symptom burden over the induction therapy.

## Figures and Tables

**Table 1:** Dose de-escalation in phase I of the trial testing siltuximab (CNTO 328) in combination with lenalidomide, bortezomib and dexamethasone

*Dose level*	*Treatment*
Level 1	Lenalidomide 25 mg orally daily on days 1–14 followed by 7-day rest every 21 days
	Bortezomib 1.3 mg/m^2^ subq/iv on days 1, 4, 8 and 11
	Dexamethasone 20 mg orally daily on days 1, 2, 4, 5, 8, 9, 11 and 12
	Siltuximab iv 11 mg/kg on day 1
Level −1	Lenalidomide 25 mg orally daily on days 1–14 followed by 7-day rest every 21 days
	Bortezomib 1.3 mg/m^2^ subq/iv on days 1, 4, 8 and 11
	Dexamethasone 20 mg orally daily on days 1, 2, 4, 5, 8, 9, 11 and 12
	Siltuximab iv 8.3 mg/kg day 1

**Table 2:** Baseline demographic and clinical characteristics of patients with newly-diagnosed, previously untreated multiple myeloma enrolled in phase I study of siltuximab in combination with RVD (*N*=11)

*Characteristic*	*No.*	*%*
*Age*		
⩽65 years	7	63.6
>65 years	4	36.4
		
*Sex*		
Male	4	36.4
Female	7	63.6
		
*Race*		
Caucasian	9	81.8
African American	2	18.2
		
*ISS stage*		
I	7	63.6
II	2	18.2
III	2	18.2
		
*ECOG performance score*		
0	6	54.5
1	5	45.5
		
*Cytogenetics karyotype*		
Normal/Diploid	4	36.4
Loss/del 13^a^	2	18.2
Hyperdiploid^b^	2	18.2
t(11;14)	1	9.1
Other	1	18.2
Unknown	1	9.1

Abbreviations: ECOG, Eastern Cooperative Oncology Group; ISS, International Staging System for multiple myeloma; RVD, lenalidomide, bortezomib, dexamethasone.

a Includes one in combination with del 17p.

b Alone or in combination with other cytogenetic abnormality.

**Table 3:** Hematological and non-hematological adverse events in patients with newly-diagnosed, previously untreated multiple myeloma during 47 cycles of therapy with siltuximab in combination with RVD (*N*=11)

*Hematological toxicity type*	*Grade*							
	*1*		*2*		*3*		*4*	
	*No.*	*%*	*No.*	*%*	*No.*	*%*	*No.*	*%*
Neutropenia	1	9.1	1	9.1	6	54.5	3	16.1
Thrombocytopenia	5	45.5	1	9.1	4	36.4	**1**	9.1
Lymphopenia	2	18.2	1	9.1	7	63.6		
Leukopenia	2	18.2	2	18.2	7	63.6		
Anemia	6	54.5	3	27.3	2	18.2		
Leukocytosis					1	9.1		

*Non-hematological toxicity type*	*Grade*			
	*2*		*3*	
	*No.*	*%*	*No.*	*%*
Nausea	4	36.4	2	18.2
Edema: Limbs	1	9.1	2	18.2
Diarrhea	3	27.3	1	9.1
Peripheral sensory neuropathy	1	9.1	1	9.1
Maculo-papular rash	1	9.1	1	9.1
Pneumonia			**1**	9.1
Fatigue	7	63.6		
Constipation	6	54.5		
Paresthesia	5	45.5		
Myalgia	4	56.4		
Dizziness	3	27.3		
Fracture	2	18.2		
Upper respiratory infection	2	18.2		
Dyspnea	2	18.2		
Vomiting	2	18.2		
Blurred vision	2	18.2		
Allergic reaction	1	9.1		
Oral mucositis	1	9.1		
Dyspepsia	1	9.1		
Dysgeusia	1	9.1		
Dehydration	1	9.1		
Pain: Back	1	9.1		
Pain: Extremity	1	9.1		
Pain: Generalized	1	9.1		
Insomnia	1	9.1		
Head boils	1	9.1		

Abbreviation: RVD, lenalidomide, bortezomib, dexamethasone.

**Table 4:** Mobilization therapy and cell characteristics in patients with newly-diagnosed, previously untreated multiple myeloma enrolled in phase I study of siltuximab in combination with RVD before ASCT (*N*=9)

*Patient number*	*Mobilization regimen*	*Number of sessions needed to harvest cells*	*Total nucleated cells collected (e8/kg)*	*Total stem cells (CD34+) collected (e6/kg)*	*Days between completion of the last treatment cycle and ASCT*	*Day of engraftment post ASCT*	*Disease progression*	*Progression-free survival (days)*
2	Plerixafor/G-CSF	6	8.01	4.16	39	12	N	862
3	Plerixafor/G-CSF	6	8.77	4.55	43	12	N	811
4	Cyclophosphamide/G-CSF	1	3.69	19.22	64	11	N	757
5	G-CSF	5	6.42	2.88	88	11	N	736
7	G-CSF	1	3.37	5.36	56	11	N	725
8	Modified CVAD/G-CSF	1	3.49	9.21	47	11	N	548
9	Plerixafor/G-CSF	5	8.46	3.46	46	11	N	717
10	G-CSF	5	9.24	4.05	39	12	N	203
11	Plerixafor/G-CSF	2	5.92	4.08	52	11	N	533

Abbreviations: ACST, autologous stem cell transplantation; CVAD, cyclophosphamide/vincristine/doxorubicin/dexamethasone; G-CSF, granulocyte colony stimulating factor; RVD, lenalidomide, bortezomib, dexamethasone.

**Table 5:** Treatment dose level, number of cycles administered and full response assessment in patients with newly-diagnosed, previously untreated multiple myeloma enrolled in phase I study of siltuximab in combination with RVD (*N*=11)

*Patient number*	*Dose level*	*Number of cycles completed*	*Response after 4 cycles*	*ASCT*	*Response after ASCT at*	
					3 mo.	6 mo.
1	1	9	VGPR (near CR^a^)	No (Consent withdrawn—patient moved out of state)		
2	1	4	VGPR	Yes	Near CR	Near CR
3	1	4	PR	Yes	VGPR	VGPR
4	1	4	PR	Yes	PR	PR
5	1	4	PR	Yes	PR	PR
6	1	3	VGPR^b^	No (Consent withdrawn due to peripheral neuropathy with pain and edema)		
7	−1	4	PR	Yes	PR	PR
8	−1	5	VGPR	Yes	Near CR	CR
9	−1	4	VGPR	Yes	VGPR	Near CR
10	−1	3	CR^b^	Yes	Lost to follow-up	
11	−1	4	MR	Yes	CR	VGPR

Abbreviations: ACST, autologous stem cell transplantation; CR, complete remission; MR, minor response; PR, partial response; RVD, lenalidomide, bortezomib, dexamethasone; VGPR, very good partial response.

a Full disease response achieved after 8 cycles of therapy.

b Full disease response achieved after 3 cycles of therapy.
